# Flavone acetic acid (FAA) with recombinant interleukin-2 (TIL-2) in advanced malignant melanoma. II: Induction of nitric oxide production.

**DOI:** 10.1038/bjc.1992.346

**Published:** 1992-10

**Authors:** L. L. Thomsen, B. C. Baguley, G. J. Rustin, S. M. O'Reilly

**Affiliations:** Cancer Research Laboratory, University of Auckland School of Medicine, New Zealand.

## Abstract

Plasma samples were collected from 20 patients undergoing phase I clinical trial with flavone-8-acetic acid (FAA; 4.8 g m-2 per dose) in combination with recombinant human interleukin-2 (rhIL-2; 6-18 i.u. m-2 per day) for the treatment of metastatic melanoma. Samples were analysed for nitrate content as an indication of the oxidation of L-arginine to nitric oxide. Pretreatment plasma nitrate levels (53 +/- 4 microM) were significantly above those of healthy volunteers (19 +/- 4 microM). The maximum plasma nitrate concentration obtained after treatment, 190 +/- 29 microM (range 49 to 655 microM), was comparable to that of mice treated with FAA. Most of the increases occurred 3-5 days after initiation of a 5 day infusion of rhIL-2, but three of the increases occurred within 2 days of a 1 h infusion of FAA alone. The maximum plasma nitrate concentrations of the three patients which underwent remission (two complete, one partial) following treatment (368 +/- 143 microM) were significantly higher (P < 0.05) than those of patients with progressive disease. Hypotension was the major dose-limiting side effect, and there was no relationship between the degree of hypotension and the rise in plasma nitrate. The results provide evidence that treatment of patients with FAA and rhIL-2 induce the synthesis of nitric oxide, a physiological mediator and potential cytotoxic agent.


Br. J. Cancer (1992), 66, 723–727 © Macmillan Press Ltd., 1992

### Flavone Acetic Acid (FAA) with recombinant interleukin-2 (TIL-2) in advanced malignant melanoma II: induction of nitric oxide production

L.L. Thomsen[1], B.C. Baguley[1], G.J.S. Rustin[2] & S.M. O'Reilly[2]

[1]*Cancer Research Laboratory, University of Auckland School of Medicine, Auckland, New Zealand;* [2]*Department of Medical Oncology, Charing Cross Hospital, Fulham Palace Road, London W6 8RF, UK.*

**Summary** Plasma samples were collected from 20 patients undergoing phase I clinical trial with flavone-8-acetic acid (FAA; 4.8 g m$^{-2}$ per dose) in combination with recombinant human interleukin-2 (rhIL-2; 6–18 i.u. m$^{-2}$ per day) for the treatment of metastatic melanoma. Samples were analysed for nitrate content as an indication of the oxidation of L-arginine to nitric oxide. Pretreatment plasma nitrate levels (53 ± 4 μM) were significantly above those of healthy volunteers (19 ± 4 μM). The maximum plasma nitrate concentration obtained after treatment, 190 ± 29 μM (range 49 to 655 μM), was comparable to that of mice treated with FAA. Most of the increases occurred 3–5 days after initiation of a 5 day infusion of rhIL-2, but three of the increases occurred within 2 days of a 1 h infusion of FAA alone. The maximum plasma nitrate concentrations of the three patients which underwent remission (two complete, one partial) following treatment (368 ± 143 μM) were significantly higher ($P<0.05$) than those of patients with progressive disease. Hypotension was the major dose-limiting side effect, and there was no relationship between the degree of hypotension and the rise in plasma nitrate. The results provide evidence that treatment of patients with FAA and rhIL-2 induce the synthesis of nitric oxide, a physiological mediator and potential cytotoxic agent.

Flavone-8-acetic acid (FAA), a synthetic flavonoid (Atassi *et al.*, 1985), has shown outstanding activity against murine experimental solid tumours (Plowman *et al.*, 1986; O'Dwyer *et al.*, 1987). This activity is augmented in some tumours by co-administration of interleukin-2 (IL-2) (Wiltrout *et al.*, 1988). However, clinical trials of FAA, administered either as a single agent or in combination with IL-2 (Kaye *et al.*, 1990), have so far not shown evidence of activity, raising the question of whether the action of FAA is species specific. One method of resolving this question is to determine whether any of the biological responses induced by FAA in mice can be detected in humans.

Studies in our laboratory have provided evidence that FAA stimulates L-arginine-dependent nitric oxide production *in vitro* by activated murine macrophages (Thomsen *et al.*, 1990). Enhanced nitrate concentration in plasma is largely due to increased biosynthesis of nitric oxide via the L-arginine-dependent pathway (Leaf *et al.*, 1990). Our finding of increased plasma nitrate concentrations in the plasma of normal and tumour-bearing mice following FAA administration indicated that this compound also stimulated nitric oxide production *in vivo* (Thomsen *et al.*, 1991). Moreover, for a series of xanthenone-4-acetic acid (XAA) analogues of FAA, the measured increase in plasma nitrate concentration was related to the tumour growth delay, suggesting that nitric oxide production was correlated in some way with the antitumour effects of FAA. Since plasma nitrate concentrations are simple to measure, it is of interest to determine whether such a response is evident in patients.

In this study, we have measured nitrate concentration in plasma samples from a series of 20 patients with metastatic malignant melanoma, taken before and after treatment with a combination of FAA and IL-2, details of which will be reported in a future article (O'Reilly *et al.*, unpublished). Three of these patients have responded to this treatment, allowing a comparison of plasma nitrate levels between responders and nonresponders.

Correspondence: G.J.S. Rustin.
Received 6 December 1991; and in revised form 5 May 1992.

### Materials and methods

### Clinical methods

Twenty patients with metastatic melanoma were entered into a phase I clinical trial of FAA (Lipha Lyonnaise Industrielle, Lyons, France) combined with rhIL-2 (Proleukin, Eurocetus, Amsterdam, Netherlands). Prior to treatment, all patients had progressive disease and a performance status $\leqslant 3$ on a 5-grade scale according to WHO criteria (Miller *et al.*, 1981).

The treatment protocol, which had been approved by the local ethical committee on human experimentation, was modified during the trial to reduce the severity of side effects (i.e. hypotension). Thus, for patients 1–8, FAA (4.8 g m$^{-2}$) was given as a 1 h infusion in 500 ml 0.9% saline without urine alkalinisation on days 1, 8, and 15. rIL-2 ($6-18 \times 10^6$ international units/m$^2$/day) was given as a continuous infusion on days 8–12 and 15–19. For patients 9–20, FAA and rhIL-2 were given as described except that rIL-2 was given on days 8–12 only.

Following treatment, patients were assessed for clinical responses according to standard WHO criteria (Miller *et al.*, 1981). Each patient received a second course of treatment after 2 weeks unless a complete response to treatment or evidence of disease progression was observed.

### Plasma samples for nitrate analyses

Blood samples, taken before and at intervals after FAA and rIL-2 administration, were collected into lithium heparin tubes. After centrifugation plasma was removed and immediately frozen at −20°C. Samples were transported between the UK and New Zealand after filter-sterilisation using cellulose acetate filters. Plasma samples were also obtained in New Zealand from blood donated by six healthy volunteers. Control experiments, in which the latter samples were stored at room temperature for up to 10 days, showed that these storage conditions did not significantly change nitrate concentrations.

Plasma nitrate levels were determined as previously described (Thomsen *et al.*, 1991). After precipitation of plasma proteins with 30% $ZnSO_4$ and reduction of nitrate in the supernatant to nitrite using acid-washed cadmium powder, nitrite was measured using a microplate assay based on the Griess reaction (Green et al., 1982). Plasma nitrate concentrations were expressed as the mean ± s.e.m. (standard error of the mean) for groups of data. Student's t-test was used to compare plasma nitrate concentrations between groups. Values for $P<0.05$ were considered significantly different.

*Monitoring of blood pressure*

Systolic and diastolic blood pressure levels were recorded at the same time as blood samples were collected from each patient during treatment.

### Results

For the 20 patients entered in the trial, pretreatment concentrations of nitrate in plasma prior to administration of FAA and rIL-2 were significantly higher than concentrations in plasma from healthy volunteers ($P<0.05$) (Table I). Plasma nitrate concentrations increased during treatment (Figure 1) with maximal concentrations observed for the patient group significantly higher than the pretreatment values ($P<0.05$) (Table I).

Increases were most commonly observed during or following rIL-2 infusion, although a substantial increase was also observed for three patients after infusion of FAA alone. Patient one showed an increase from a pretreatment level of 40 μM to 655 μM at 18 h after the first infusion of FAA during the first treatment course; patient 10 showed an increase from 70 μM to 323 μM at 24 h after the third infusion of FAA in the second treatment course; patient 17 showed an increase from 82 μM to 195 μM at 24 h after the first infusion of FAA during the second treatment course (Figure 1).

Three of the 20 patients responded to the treatment regime. Patients one and two showed complete responses and patient 11 showed a partial response. Maximal plasma nitrate concentrations were significantly greater for these three patients compared with the 17 patients with progressive disease following treatment ($P<0.05$) (Table II). A drop of systolic blood pressure of >30 mmHg which required intravenous fluid therapy occurred after 22 of 97 infusions of FAA. There was no relationship between plasma nitrate levels and blood pressure.

**Table I** Plasma nitrate concentrations (mean ± s.e.m.) in healthy volunteers and cancer patients before and after treatment

| *Group* | *Plasma nitrate* (μM) |
|---|---|
| Healthy volunteers | 19 ± 4[a] |
| Cancer patients – before treatment | 53 ± 4 |
| Cancer patients – after treatment[b] | 190 ± 29[a] |

[a]$P<0.05$ compared with patients' pretreatment value; [b]Maximal concentration obtained from plasma samples analysed for each patient.

**Figure 1** Plasma nitrate concentrations for patients before and during treatment with FAA in combination with rIL-2. O and Δ are the first and second courses of treatment respectively.

**Table II** A comparison between plamsa nitrate concentrations observed for patients showing a clinical response to treatment ($n = 3$) and those with progressive disease after treatment ($n = 17$). Plasma nitrate levels are the maximal concentrations (mean ± s.e.m.) for each patient group

| *Cancer patients* | *Plasma nitrate (μM)* |
|---|---|
| Progressive disease | 158 ± 18 |
| Clinical response | 368 ± 143[a] |

[a]$P < 0.05$ compared with progressive disease.

### Discussion

The present study appears to be the first to document increased plasma nitrate levels in humans in response to a clinical treatment protocol incorporating FAA and IL-2, although an plasma nitrate increases following administration of IL-2 alone have been reported recelty (Hibbs *et al.*, 1992). In 15 of the 20 patients studied here, there was at least a doubling of the pretreatment plasma nitrate concentration. The maximum plasma nitrate concentrations obtained after treatment ranged from 49 to 655 μM (mean 190 μM). This compares with the maximum plasma nitrate concentrations 12 h after a therapeutic dose of FAA of 85 μM (non tumour-bearing mice) and 630 μM (colon 38 tumour-bearing mice) (Thomsen *et al.*, 1991). Most of the increases in plasma nitrate concentration in patients was observed 3–5 days after commencement of infusion of IL-2, suggesting that IL-2 may be an important stimulator of nitric oxide production in humans treated with FAA and IL-2 combination therapy. Clinical studies show that IL-2 induces increased serum concentrations of the cytokines TNF-α and IFN-γ (Gemlo *et al.*, 1988; Blay *et al.*, 1990; Boccoli *et al.*, 1990). These cytokines are known to induce nitric oxide synthesis in both macrophages and endothelial cells in experimental systems (Kilbourn & Belloni, 1990). Increases in plasma nitrate may thus be mediated by an IL-2-induced cytokine cascade.

Twelve of the patients in this study also had plasma levels of TNF, GM-CSF, and IL-6 measured (Haworth *et al.*, unpublished). Interestingly a very marked rise in all these cytokines was noted 2–8 h after a course of FAA, but only when the FAA was given 2–4 days after infusion of IL-2. The highest nitrate level was found at a similar time point in 11 of the 14 patients who had nitrate measured at that time.

Substantial increases in plasma nitrate levels were observed in three patients within 2 days of infusion of FAA alone, suggesting that stimulation of nitric oxide production is not exclusively dependent on infusion of IL-2. In mice, FAA stimulates increases in plasma nitrate over a period of 12 h (Thomsen *et al.*, 1991), and induces increased serum concentrations of the cytokines TNF-α and IFN-γ (Urba *et al.*, 1988; Mace *et al.*, 1990) suggesting that nitric oxide production may be induced either directly by FAA or indirectly through the induction of cytokines. It is therefore possible that, in these three patients, FAA is having a biological effect similar to that found in mice. Since the number of plasma samples studied was small, further studies are required to provide a definitive answer to whether FAA elevates plasma nitrate in a clinical situation. In particular, analysis of plasma samples taken 2–7 days after infusion of FAA alone, which were not available from this study, may help to clarify the effect of FAA on nitric oxide production.

Pretreatment nitrate levels ($53 \pm 4\ \mu M$) were found to be significantly above those of healthy volunteers ($19 \pm 4\ \mu M$). It is possible that this is a consequence of increased nitric oxide synthesis in response to higher basal production of cytokines in some cancer patients. Plasma TNF concentrations have been shown on average to be higher in cancer patients than in control patients (Balkwill *et al.*, 1987).

Hypotension was the dose-limiting side-effect of the combination therapy of FAA with IL-2, as it is with TNF (Creaven *et al.*, 1989) and endotoxin (Engelhardt *et al.*, 1991). Kilbourn *et al.*, 1990 and Thiemermann & Vane, 1990 have shown that nitric oxide is involved in the induction of hypotension by TNF and endotoxin in animals. We have not been able to measure blood pressure in mice treated with FAA, although the transient rise in haemoglobin concentration observed in mice treated with FAA (Ching *et al.*, 1991) is consistent with a drop in blood pressure. Studies of the relationship between blood pressure and plasma nitrate levels in mice would allow a clearer interspecies comparison of the effects of FAA and IL-2.

Despite the fact that nitric oxide is known to regulate blood pressure in humans, no correlation was found between plasma nitrate levels and blood pressure in patients treated with this clinical regime. A possible explanation is that the major source of nitrate in plasma is from sources other than the endothelial cells controlling blood pressure. Alternatively, the effect of nitric oxide on blood pressure may be counterbalanced by the release of other substances such as endothelin (Clarke *et al.*, 1989), thereby obscuring the simple relationship between nitric oxide production reflected as nitrate in plasma and hypotension.

The number of patients responding to treatment in this study (3 of 20) is too small to provide adequate statistical evaluation of the relationship between the maximum measured plasma nitrate concentration and the response rate. Nevertheless, the finding of a significant difference in nitrate concentrations between responders and non-responders ($P < 0.05$) suggests that further analysis should be carried out. In mice it has been suggested, on the basis of the correlation between nitric oxide production and antitumour activity of FAA analogues, that nitric oxide production contributes to the antitumour effect of FAA (Thomsen *et al.*, 1990; 1991). Nitric oxide may have a cytostatic and cytotoxic role as a consequence of its ability to bind to and inhibit the active iron-sulphur centres of key enzymes in ATP and DNA synthesis (Lancaster & Hibbs, 1990).

The lack of effect of FAA alone on plasma nitrate levels, which was observed in the majority of patients, suggests that its failure as a clinical antitumour agent may be a lack of dose potency of this agent in stimulating nitric oxide production in human systems. Other agents which stimulate the nitric oxide synthesis pathway, such as TNF-α (Creaven *et al.*, 1989) and endotoxin (Engelhardt *et al.*, 1991), are currently being investigated as potential clinical antitumour agents. 5,6-Dimethyl XAA, a more dose-potent analogue of FAA which is a powerful inducer of nitric oxide both *in vitro* (Thomsen et al., 1990) and *in vivo* (Thomsen *et al.*, 1991), is currently under consideration as a candidate for clinical trial. Studies of the relationship between plasma nitrate concentration and clinical response to these agents may be pivotal in establishing the direction of future investigations of host-mediated therapies. Development of more potent agents in stimulation of nitric oxide production in human systems may need to be emphasised, with nitrate analysis providing a useful predictive test for a clinical response to such treatments.

This work was supported by the Ruth Spencer Medical Research Fellowship Trust (L.L.T.); the Auckland Division of the Cancer Society of New Zealand and the Health Research Council of New Zealand (B.C.B.); the Cancer Research Campaign and a grant from Lipha Lyonnaise Industrielle (G.J.S.R. and S.O'R.). We are grateful to C. Bone, G. Brunstrom and M. Stratford for sample processing and to our research nurses N. Howells and K. Farmer.

### References

ATASSI, G., BRIET, P., BERTHELON, J.-J. & COLLONGES, F. (1985). Synthesis and antitumour activity of some 8-substituted 4-oxo-4H-1-benzopyrans. *Eur. J. Med. Chem.*, **5**, 393–402.

BALKWILL, F.R., BURKE, F., TALBOT, D. & 5 others (1987). Evidence for tumour necrosis factor/cachexia production in cancer. *Lancet*, **ii**, 1229–1234.

BLAY, J.-Y., FARROT, M.C., NEGRIER, S. & 6 others (1990). Correlation between clinical response to interleukin 2 therapy and sustained production of tumor necrossis factor. *Cancer Res.*, **50**, 2371–2374.

BOCCOLI, G., MASCIULLI, R., RUGGERI, E.M. & 8 others (1990). Adoptive immunotherapy of human cancer – the cytokine cascade and monocyte activation following high-dose interleukin-2 bolus treatment. *Cancer Res.*, **50**, 5795–5800.

CHING, L.M., MCKEAGE, M.J., JOSEPH, W.R., KESTELL, P., ZWI, L.J. & BAGULEY, B.C. (1991). Haematological effects in mice of the antitumour agents xanthenone-4-acetic acid, 5,6-methyl-xanthenone-4-acetic acid and flavone acetic acid. *Cancer Chemother. Pharmacol.*, **28**, 414–419.

CLARKE, J.G., BENJAMIN, N., LARKIN, S.W., WEBB, D.J., DAVIES, G.J. & MASERI, A. (1989). Endothelin is a potent long-lasting vasoconstrictor in men. *Am. J. Physiol.*, **257**, H2033–H2035.

CREAVEN, P.J., BRENNER, D.E., COWENS, J.W. & 6 others (1989). A phase I clinical trial of recombinant human tumor necrosis factor given daily for five days. *Cancer Chemother. Pharmacol.*, **23**, 186–191.

ENGLEHARDT, R., MACKENSEN, A. & GALANOS, C. (1991). Phase-I trial of intravenously administered endotoxin (*Salmonella abortus equi*) in cancer patients. *Cancer Res.*, **51**, 2524–2530.

GEMLO, B.T., PALLADINO, M.A., JAFFE, H.S., ESPEVIK, T.P. & RAYNOR, A.A. (1988). Circulating cytokines in patients with metastatic cancer treated with recombinant interleukin 2 and lymphokine-activated killer cells. *Cancer Res.*, **48**, 5864–5867.

GREEN, L.C., WAGNER, D.A., GLOGOWSKI, J., SKIPPER, P.L., WISHNOK, J.S. & TANNEBAUM, S.R. (1982). Analysis of nitrate, nitrite, and ($^{15}$N)nitrate in biological fluids. *Anal. Biochem.*, **126**, 131–138.

HIBBS, J.B., WESTENFELDER, C., TAINTOR, R. & 8 others (1992). Evidence for cytokine-inducible nitric oxide synthesis from L-arginine in patients receiving interleukin-2 therapy. *J. Clin. Invest.*, **89**, 867–877.

KAYE, S.B., CLAVEL, M., DODION, P. & 9 others (1990). Phase-II trials with flavone acetic acid (NSC-347512, LM975) in patients with advanced carcinoma of the breast, colon, head and neck and melanoma. *Inv. New. Dr.*, **8**, S95–S99.

KILBOURN, R.G. & BELLONI, P. (1990). Endothelial cell production of nitrogen oxides in response to interferon-gamma in combination with tumor necrosis factor, interleukin-1, or endotoxin. *J. Natl Cancer Inst.*, **82**, 772–776.

KILBOURN, R.G., GROSS, S.S., JUBRAN, A. & others (1990). $N^G$-methyl-L-arginine inhibits tumor necrosis factor-induced hypotension – implications for the involvement of nitric oxide. *Proc. Natl Acad. Sci. USA*, **87**, 3629–3632.

LANCASTER, J.R. & HIBBS, J.B. (1990). EPR demonstration of iron nitrosyl complex formation by cytotoxic activated macrophages. *Proc. Natl Acad. Sci. USA*, **87**, 1223–1227.

LEAF, C.D., WISHNOK, J.S., HURLEY, J.P., ROSENBLAD, W.D., FOX, J.G. & TANNENBAUM, S.R. (1990). Nitrate biosynthesis in rats, ferrets and humans - precursor studies with L-arginine. *Carcinogenesis*, **11**, 855-858.

MACE, K.F., HORNUNG, R.L., WILTROUT, R.H. & YOUNG, H.A. (1990). Correlation between *in vivo* induction of cytokine gene expression by flavone acetic acid and strict dose dependency and therapeutic efficacy against murine renal cancer. *Cancer Res.*, **50**, 1742-1747.

MILLER, A.B., HOOGSTRATEN, B., STAQUET, M. & WINKLER, A. (1981). Reporting results of cancer treatment. *Cancer*, **1981**, 207-214.

O'DWYER, P.J., SHOEMAKER, D., ZAHARKO, S. & 8 others (1987). Flavone acetic acid (LM 975, NSC 347512), a novel antitumor agent. *Cancer Chemother. Pharmacol.*, **19**, 6-10.

PLOWMAN, J., NARYANAN, V.L., DYKES, D. & 4 others (1986). Flavone acetic acid: a novel agent with preclinical antitumor activity against colon adenocarcinoma 38 in mice. *Cancer Treat. Rep.*, **70**, 631-638.

THIEMERMANN, C. & VANE, J. (1990). Inhibition of nitric oxide synthesis reduces the hypotension induced by bacterial lipopolysaccharides in the rat *in vivo*. *Eur. J. Pharmacol.*, **182**, 591-595.

THOMSEN, L.L., CHING, L.M. & BAGULEY, B.C. (1990). Evidence for the production of nitric oxide by activated macrophages treated with the antitumor agents flavone-8-acetic acid and xanthenone-4-acetic acid. *Cancer Res.*, **50**, 6966-6970.

THOMSEN, L.L., CHING, L.M., ZHUANG, L., GAVIN, J.B. & BAGULEY, B.C. (1991). Tumor-dependent increased plasma nitrate concentrations as an indication of the antitumor effect of flavone-8-acetic acid and analogues in mice. *Cancer Res.*, **51**, 77-81.

URBA, W.J., LONGO, D.L., LOMBARDO, F.A. & WEISS, R.B. (1988). Enhancement of natural killer activity in human peripheral blood by flavone acetic acid. *J. Natl Cancer Inst.*, **80**, 521-525.

WILTROUT, R.H., BOYD, M.R., BACK, T.C., SALUP, R.R., ARTHUR, J.A. & HORNUNG, R.L. (1988). Flavone-8-acetic acid augments systemic natural killer cell activity and synergizes with IL-2 for treatment of murine renal cancer. *J. Immunol.*, **140**, 3261-3265.


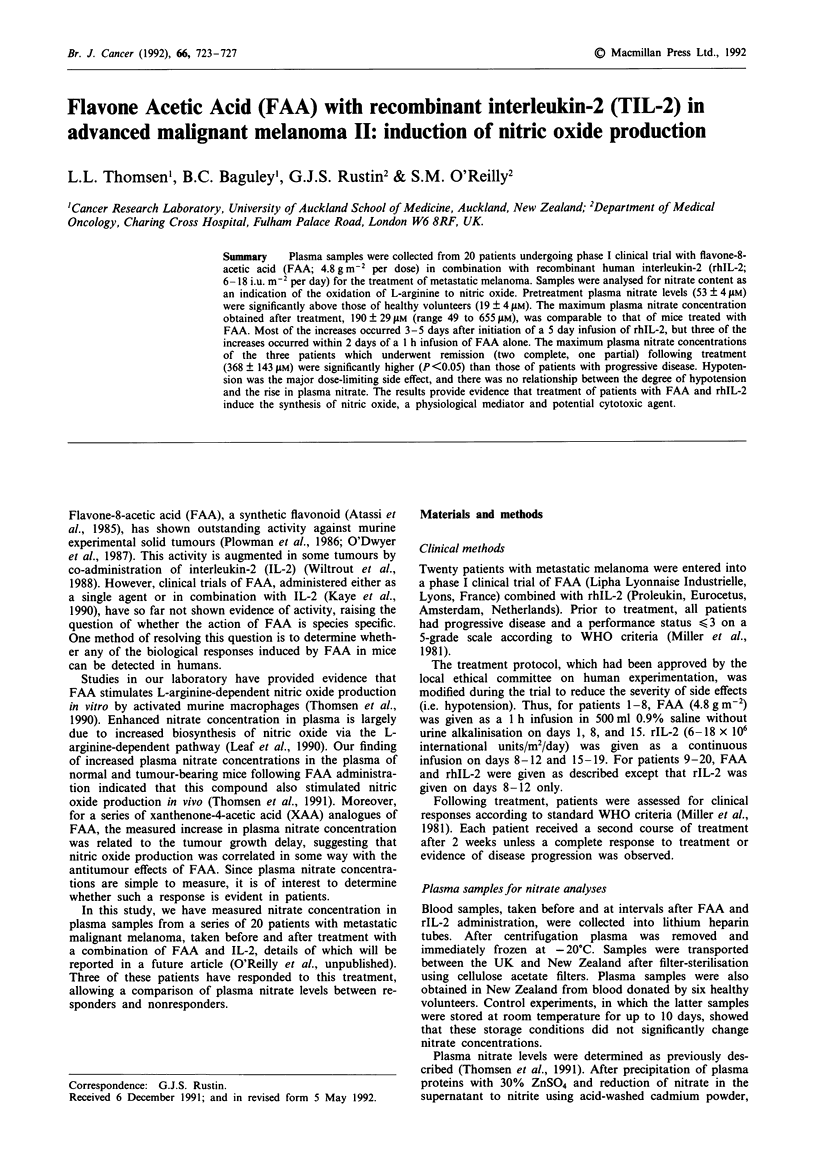

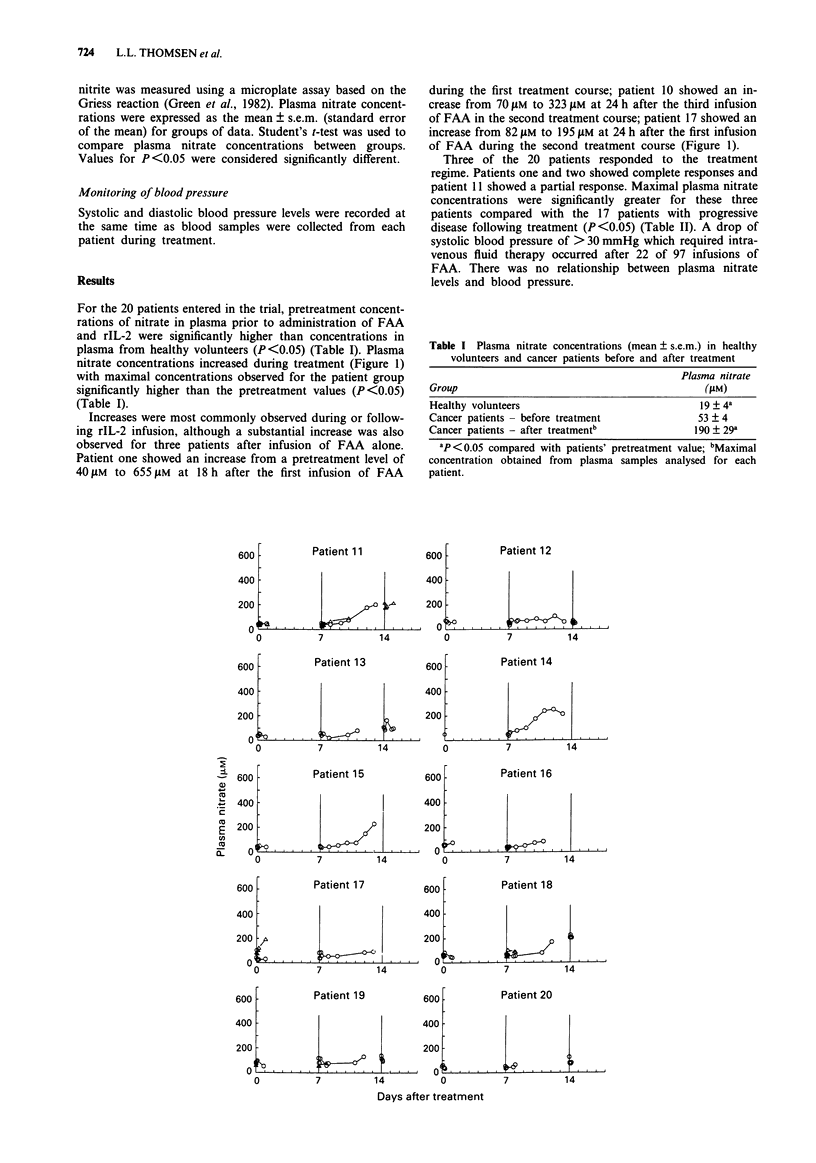

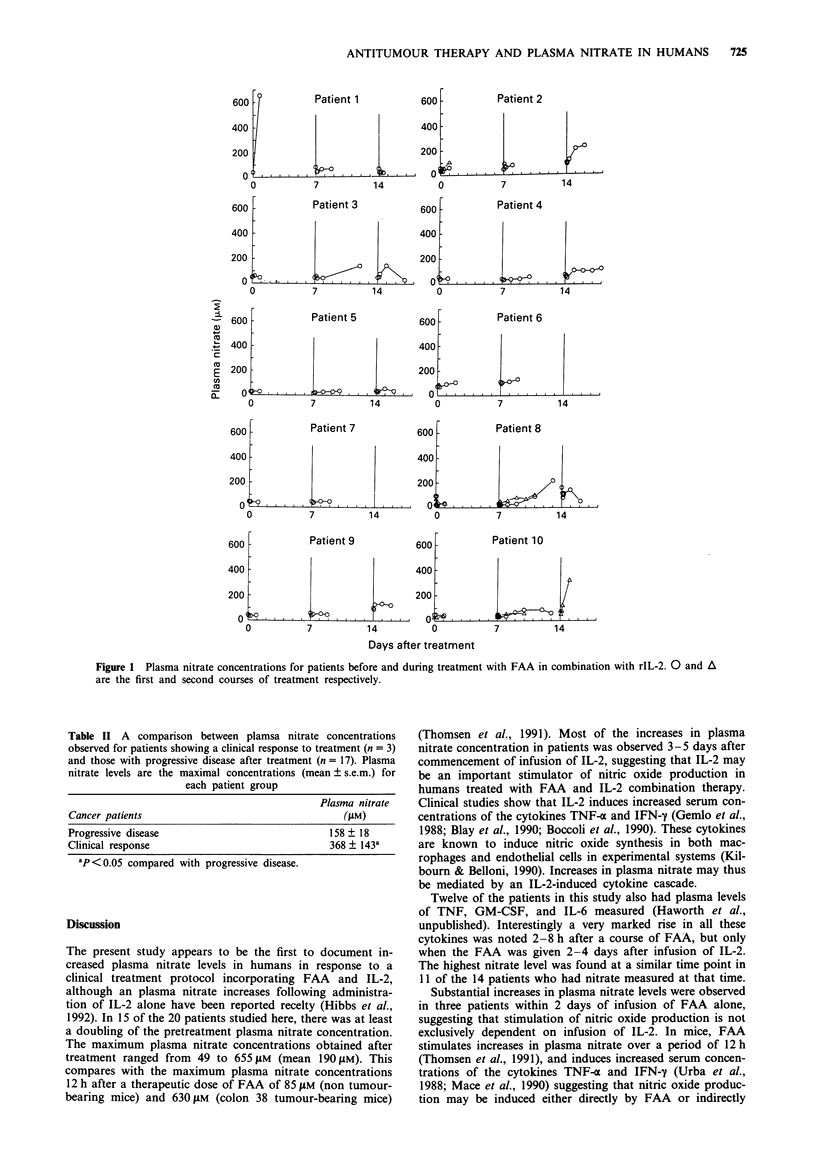

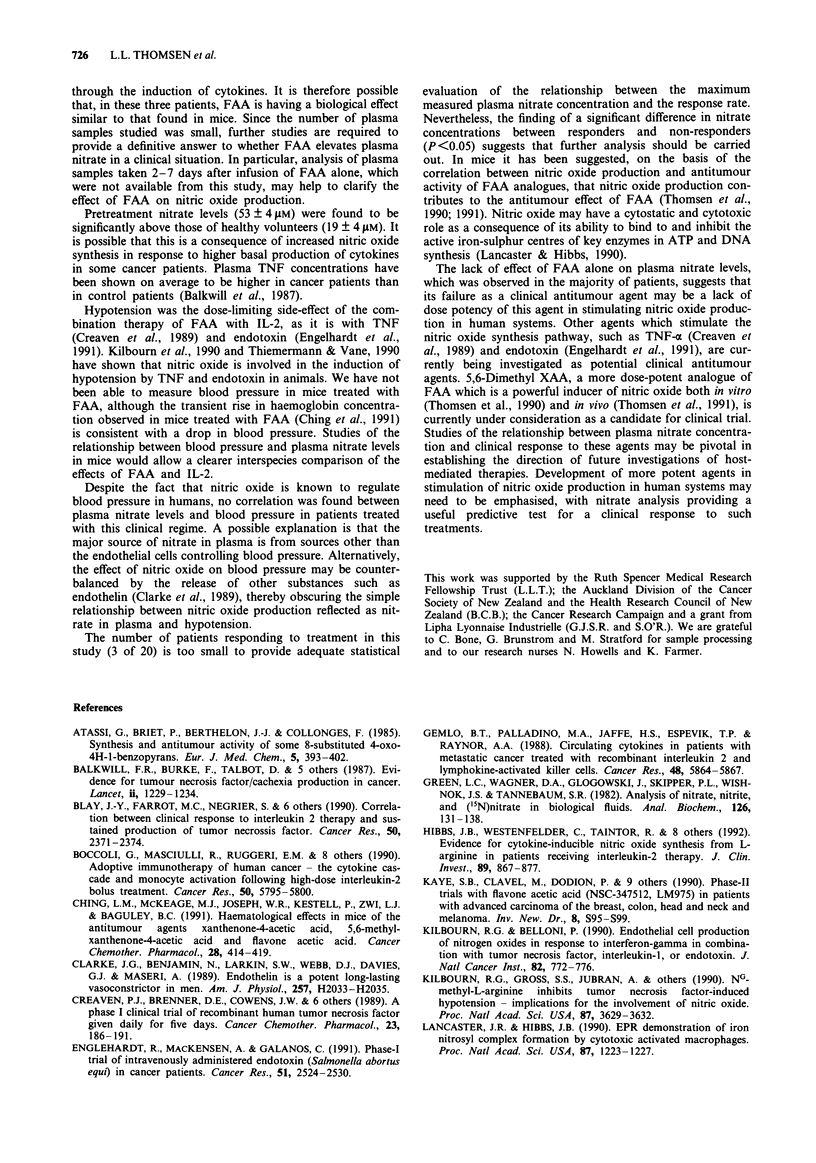

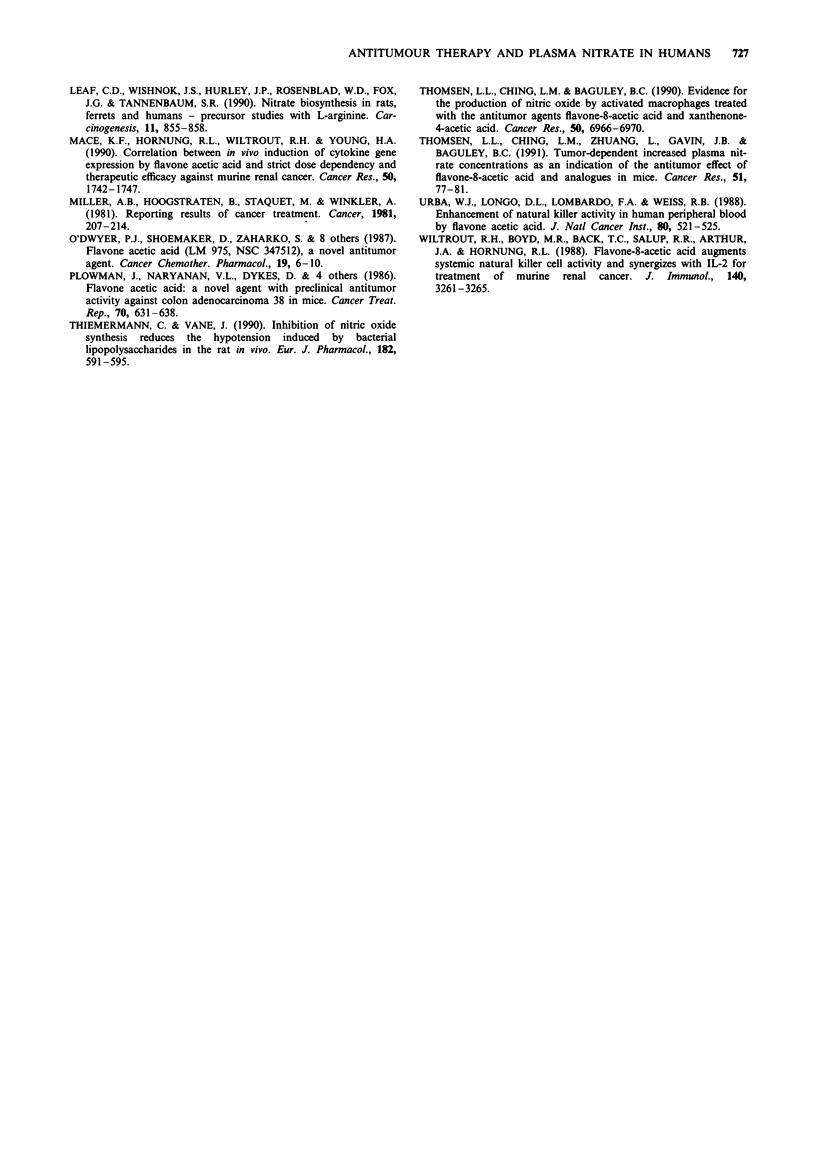


## References

[OCR_00570] Balkwill F., Osborne R., Burke F., Naylor S., Talbot D., Durbin H., Tavernier J., Fiers W. (1987). Evidence for tumour necrosis factor/cachectin production in cancer.. Lancet.

[OCR_00573] Blay J. Y., Favrot M. C., Negrier S., Combaret V., Chouaib S., Mercatello A., Kaemmerlen P., Franks C. R., Philip T. (1990). Correlation between clinical response to interleukin 2 therapy and sustained production of tumor necrosis factor.. Cancer Res.

[OCR_00579] Boccoli G., Masciulli R., Ruggeri E. M., Carlini P., Giannella G., Montesoro E., Mastroberardino G., Isacchi G., Testa U., Calabresi F. (1990). Adoptive immunotherapy of human cancer: the cytokine cascade and monocyte activation following high-dose interleukin 2 bolus treatment.. Cancer Res.

[OCR_00585] Ching L. M., McKeage M. J., Joseph W. R., Kestell P., Zwi L. J., Baguley B. C. (1991). Haematological effects in mice of the antitumour agents xanthenone-4-acetic acid, 5,6-dimethyl-xanthenone-4-acetic acid [correction of 5,6-methyl-] and flavone acetic acid.. Cancer Chemother Pharmacol.

[OCR_00592] Clarke J. G., Benjamin N., Larkin S. W., Webb D. J., Davies G. J., Maseri A. (1989). Endothelin is a potent long-lasting vasoconstrictor in men.. Am J Physiol.

[OCR_00597] Creaven P. J., Brenner D. E., Cowens J. W., Huben R. P., Wolf R. M., Takita H., Arbuck S. G., Razack M. S., Proefrock A. D. (1989). A phase I clinical trial of recombinant human tumor necrosis factor given daily for five days.. Cancer Chemother Pharmacol.

[OCR_00603] Engelhardt R., Mackensen A., Galanos C. (1991). Phase I trial of intravenously administered endotoxin (Salmonella abortus equi) in cancer patients.. Cancer Res.

[OCR_00608] Gemlo B. T., Palladino M. A., Jaffe H. S., Espevik T. P., Rayner A. A. (1988). Circulating cytokines in patients with metastatic cancer treated with recombinant interleukin 2 and lymphokine-activated killer cells.. Cancer Res.

[OCR_00616] Green L. C., Wagner D. A., Glogowski J., Skipper P. L., Wishnok J. S., Tannenbaum S. R. (1982). Analysis of nitrate, nitrite, and [15N]nitrate in biological fluids.. Anal Biochem.

[OCR_00620] Hibbs J. B., Westenfelder C., Taintor R., Vavrin Z., Kablitz C., Baranowski R. L., Ward J. H., Menlove R. L., McMurry M. P., Kushner J. P. (1992). Evidence for cytokine-inducible nitric oxide synthesis from L-arginine in patients receiving interleukin-2 therapy.. J Clin Invest.

[OCR_00626] Kaye S. B., Clavel M., Dodion P., Monfardini S., ten Bokkel-Huinink W., Wagener D. T., Gundersen S., Stoter G., Smith J., Renard J. (1990). Phase II trials with flavone acetic acid (NCS. 347512, LM975) in patients with advanced carcinoma of the breast, colon, head and neck and melanoma.. Invest New Drugs.

[OCR_00632] Kilbourn R. G., Belloni P. (1990). Endothelial cell production of nitrogen oxides in response to interferon gamma in combination with tumor necrosis factor, interleukin-1, or endotoxin.. J Natl Cancer Inst.

[OCR_00638] Kilbourn R. G., Gross S. S., Jubran A., Adams J., Griffith O. W., Levi R., Lodato R. F. (1990). NG-methyl-L-arginine inhibits tumor necrosis factor-induced hypotension: implications for the involvement of nitric oxide.. Proc Natl Acad Sci U S A.

[OCR_00644] Lancaster J. R., Hibbs J. B. (1990). EPR demonstration of iron-nitrosyl complex formation by cytotoxic activated macrophages.. Proc Natl Acad Sci U S A.

[OCR_00651] Leaf C. D., Wishnok J. S., Hurley J. P., Rosenblad W. D., Fox J. G., Tannenbaum S. R. (1990). Nitrate biosynthesis in rats, ferrets and humans. Precursor studies with L-arginine.. Carcinogenesis.

[OCR_00657] Mace K. F., Hornung R. L., Wiltrout R. H., Young H. A. (1990). Correlation between in vivo induction of cytokine gene expression by flavone acetic acid and strict dose dependency and therapeutic efficacy against murine renal cancer.. Cancer Res.

[OCR_00664] Miller A. B., Hoogstraten B., Staquet M., Winkler A. (1981). Reporting results of cancer treatment.. Cancer.

[OCR_00669] O'Dwyer P. J., Shoemaker D., Zaharko D. S., Grieshaber C., Plowman J., Corbett T., Valeriote F., King S. A., Cradock J., Hoth D. F. (1987). Flavone acetic acid (LM 975, NSC 347512). A novel antitumor agent.. Cancer Chemother Pharmacol.

[OCR_00674] Plowman J., Narayanan V. L., Dykes D., Szarvasi E., Briet P., Yoder O. C., Paull K. D. (1986). Flavone acetic acid: a novel agent with preclinical antitumor activity against colon adenocarcinoma 38 in mice.. Cancer Treat Rep.

[OCR_00680] Thiemermann C., Vane J. (1990). Inhibition of nitric oxide synthesis reduces the hypotension induced by bacterial lipopolysaccharides in the rat in vivo.. Eur J Pharmacol.

[OCR_00686] Thomsen L. L., Ching L. M., Baguley B. C. (1990). Evidence for the production of nitric oxide by activated macrophages treated with the antitumor agents flavone-8-acetic acid and xanthenone-4-acetic acid.. Cancer Res.

[OCR_00692] Thomsen L. L., Ching L. M., Zhuang L., Gavin J. B., Baguley B. C. (1991). Tumor-dependent increased plasma nitrate concentrations as an indication of the antitumor effect of flavone-8-acetic acid and analogues in mice.. Cancer Res.

[OCR_00699] Urba W. J., Longo D. L., Lombardo F. A., Weiss R. B. (1988). Enhancement of natural killer activity in human peripheral blood by flavone acetic acid.. J Natl Cancer Inst.

[OCR_00704] Wiltrout R. H., Boyd M. R., Back T. C., Salup R. R., Arthur J. A., Hornung R. L. (1988). Flavone-8-acetic acid augments systemic natural killer cell activity and synergizes with IL-2 for treatment of murine renal cancer.. J Immunol.

